# The Outrage Effect of Personal Stake, Familiarity, Effects on Children, and Fairness on Climate Change Risk Perception Moderated by Political Orientation

**DOI:** 10.3390/ijerph17186722

**Published:** 2020-09-15

**Authors:** Myoungsoon You, Youngkee Ju

**Affiliations:** 1 Department of Health Science in the Graduate School of Public Health, Seoul National University, Seoul 08826, Korea; msyou@snu.ac.kr; 2 Media School, Hallym University, Chuncheon 24252, Korea

**Keywords:** risk perception, climate change, outrage factor, media use, political orientation

## Abstract

Outrage factors are perceived characteristics of risk that provoke emotional responses and influence risk perception. Although several studies examined how multiple influences affect climate change risk perception, outrage factors have not been comprehensively assessed in the context of climate change risk perception. Using an online survey in South Korea (*n* = 592), we investigated outrage factors associated with climate change risk perception and whether political orientation moderates these outrage effects. We considered 11 of 20 outrage factors: voluntariness, controllability, familiarity, fairness, uncertainty, delayed effects, effects on children, trust, reversibility, personal stake, and human vs. natural origin. Factors that overlapped with the selected outrage factors or those that were not relevant to climate change were excluded. The survey revealed that the climate change risk perception of an individual increased when they perceived climate change to be relevant to their personal lives, when they felt unfamiliar with climate change, when they thought climate change would have a severe impact on children, or when they thought climate change would have unequal consequences. Moreover, respondents who identified as political conservatives were subject to a greater outrage effect of personal stake for climate change. The implications of the outrage effect on climate change risk perception and the greater vulnerability of conservatives to outrage effect are discussed.

## 1. Introduction

Climate change is a serious problem in many parts of the world. In a study of climate change risk perception in 119 countries, people in Europe (e.g., France, Spain, Italy, and Turkey), Africa (Mali and Tanzania), and Asia (Japan and South Korea), as well as most people in South American nations and Australia were highly aware of climate change and felt that it was a serious threat [1]. An appropriate level of climate change risk perception is critical, since mitigating climate change requires urgent reactions and risk perception that can be used as a motivating factor to promote changes in behavior that can affect climate change [2,3,4]. Recognizing the substantial importance of risk perception, in this study we examined the dynamics of climate change risk perception.

### 1.1. Affective Dimensions of Climate Change Risk Perception

Reflecting the practical significance of climate change, multiple studies have examined factors that influence climate change risk perception [2,5,6,7,8,9,10]. These influences are categorized into four dimensions: cognitive factors (e.g., knowledge), experiential factors (e.g., affect and personal experience of extreme weather), socio-cultural factors (e.g., culture, values, and worldviews), and socio-demographic factors (e.g., education, age, income, and religion) [11]. The magnitudes of these dimensions have also been examined. For example, a recent study reported that socio-cultural factors, such as biospheric environmental values and individualistic worldviews, play much greater roles than knowledge, a factor in the cognitive dimension, in guiding climate change risk perception [10].

Negative affects are often regarded as one of the single most important determinants of global warming risk perception, and can alone explain about 20–30% of the variance in climate change risk perception [2,11]. The affective dimension originates from perspectives that a good or bad affect is attached to each and every construct in our memory and is activated automatically, or requires less mental effort to influence later information processing; multiple constructs can be used to represent this affect-driven information processing, such as “affect heuristic” [12,13], “risk-as-feelings” [14], “somatic markers” [15], “affective pool” [16], “hot cognition” [17,18], “affective memory” [19], and affective tag [20]. One can expect that climate change risk perception involves these affective constructs in a way that climate information saved in the memory is associated with a positive or negative affective tag that influences the level of risk perception. This means that climate change risk perception involves a dichotomous affect which is generally described as a “faint whisper of emotion” [21], having a less concrete nature on its own relative to an actual emotion. Thus, an individual may have a vague attitude toward climate change that is good or bad.

However, it is also highly likely that individuals recognize certain characteristics of climate change (e.g., “it is uncontrollable,” “it has a catastrophic potential,” “it would have a delayed consequence,” “it is worsened by irresponsible business and governments”). The recognition of these exemplified characteristics can be a source of emotive response to climate change, which is sufficiently specific to result in dread, anger, or other emotions. These perceived risk characteristics eliciting an emotive response and heightening risk perceptions are labeled as “outrage factors” [22].

Taking an emotional approach to climate change risk perception can enrich the study of risk perception by connecting risk response to the emotional domain and leading to a more comprehensive understanding of an individuals’ risk response. Taking a closer look at the emotive dynamics of climate change risk perception, the present study investigated whether outrage factors influence climate change risk perception and which outrage factors are specifically influential in guiding risk perception. By examining an additional dimension of risk characteristics, which is likely to be emotive itself, the results of this investigation can provide a deeper understanding of the dynamics of climate change risk perception.

### 1.2. Exploring Climate Change Risk Characteristics as Outrage Factor

Several early studies examined characteristics of climate change [23,24,25,26]. For example, climate change was examined as one type of risk among 65 ecological risk items [23]. In addition, 13 global climate change (GCC) risks, such as extreme temperatures, frequent flooding events, increased rainfall, and rising sea levels were examined together with 12 non-GCC risks using factor analyses [25]. As a result, five factors (i.e., impact on species, human benefits, impact on humans, avoidability, and knowledge of impacts) or four factors (i.e., impacts, avoidability/controllability, acceptability, and understandability) were extracted. Although these results are useful in understanding risk characteristics that influence the risk perception regarding the environment in general, they do not tell us exactly what lay people think when they hear the term “climate change” since climate change was not separated from multiple non-climate change risks in the analysis. Moreover, scales to measure risk characteristics in these studies focused on environmental risks in general rather than the specified risk of climate change [23]. Here, we take an alternative approach that uses generic scales to measure climate change risk characteristics with operationalization of outrage factors as described by Covello and Sandman [22].

A common assumption of previous risk perception studies is that the public holds “a richer definition of risk,” that “incorporates a number of more qualitative characteristics” [27] (p. 303). Therefore, risk perceptions of experts and the public are generally believed to differ and the latter is subject to situational and conditional factors beyond technical or objective evaluations. Risk perception studies in the psychometric paradigm in particular are based on this assumption, and thereby investigate perceived risk characteristics as possible sources of variation in the level of risk perception between the public and experts [28,29,30,31,32].

In a similar vein, Covello and Sandman [22] define risk as hazard plus outrage, wherein hazard represents actual danger, the degree of which is determined by the estimated severity and probability of the danger, and outrage refers to an emotional response to the danger elicited by risk characteristics that are not directly related to technical evaluations of severity and probability. This perspective of risk suggests that ordinary people’s risk perception is determined not only by the technical evaluation of a risk, but by their emotive response to it. Outrage factors refer to characteristics of a risk that is perceived and elicits an emotive response, which in turn can heighten risk perception. Covello and Sandman listed 20 outrage factors that elicit an emotive response [22]. The original 20 outrage factors include voluntariness, controllability, familiarity, fairness, benefits, catastrophic potential, understanding, uncertainty, delayed effects, effects on children, effects on future generations, victim identity, dread, trust, media attention, accident history, reversibility, personal stake, ethical/moral nature, and human vs. natural origin. In this respect, the psychometric paradigm dating back to the seminal work of Gilbert White in 1945 [33] concerning natural hazards can be regarded as having further evolved into the outrage factor study paradigm, with various, undirected risk characteristics being compiled into a group that has a common trait of eliciting emotive responses. Although outrage factors are conceptually explained to elicit emotive responses, this emotive response has not been measured using either self-reporting scales or physiological measures such as heart rate and skin conductance. Studies involving the quantitative measurement of these factors to examine risk perception are needed. We consider that the outrage approach to risk perception would be more appropriate than the psychometric approach because the results of empirical research based on the former approach can be converged on a better understanding of the outrage dynamics in general, whereas multiple studies based on the latter approach would just accumulate empirical knowledge of individual risk characteristics that rarely interconnect with each other, hindering theoretical development.

Several empirical studies examined the roles of outrage factors. For example, risk perception by Americans of milk containing a recombinant bovine growth hormone was associated with involuntariness, unfamiliarity, lack of trust, and lack of tangible consumer benefits [29]. Risk perception of South Koreans of Chinese and Japanese food was associated with controllability, effects on children, inequality, dread, and benefits [34]. When risks with English labeling, such as benzopyrene in noodle soup, norovirus in school cafeteria food, and Salmonella in infant food, were presented to South Korean respondents, familiarity and catastrophic potential were added to dread and effect on children as influential outrage factors [35]. Together, these findings indicate that outrage factors play a role in risk perception regarding everyday food consumption.

The risk perception regarding an environmental issue, such as fine dust, is also subject to outrage effect. When a nationwide online survey (*n* = 1000) was conducted to measure the magnitude of 14 outrage factors associated with fine dust risk perception, personal stake, dread, moral nature, and catastrophic potential were the most significant outrage factors observed [36]. The risk perception increased among people who perceived that the environmental hazard had personal implications or was associated with fearful images and irresponsible governmental/corporate action. The risk perception was also increased when fine dust was thought to cause large scale damage to many people simultaneously. Considering the pervasiveness of outrage effects observed in cases of risks associated with foods and fine dust, examining the outrage effect on perception of the risk of climate change would also be valuable.

### 1.3. Political Orientation and Outrage Factors

In addition to examining the influence of outrage factors, investigating moderating conditions can provide a deeper understanding of the association between the main predictor and the predicted outcome [37]. Areas of further research on agenda-setting effects, for example, can include an evaluation into how the frequency of media coverage interacts with moderators (i.e., demographic, attitudinal, and behavioral factors) to influence the perceived importance of social issues [38]. Thus, an evaluation of the possible interaction effects between outrage factors and their moderators may promote a better understanding of the dynamics of climate change risk perception.

When evaluating moderators that are likely to interact with outrage factors, we considered the studies that reported the influence of political orientation on climate change risk perception [39,40,41,42]. In telephone surveys in New Hampshire and Michigan, for example, levels of concern about climate change were higher among educated Democrats compared to educated Republicans [41]. This difference raises an additional question: Will outrage factors show varying influences on climate change risk perception for liberals and conservatives? This question is based on the idea that if political orientation influences climate change risk perception, it would also be possible that the perception of outrage factors and its influence on risk perception can vary according to different political orientations. Investigating this question would inform the mechanism by which outrage effect influences climate change risk perception, and could also provide more detailed knowledge about how political orientation can guide this risk perception.

Our review of the current literature raises the following questions:

(a) Do individual outrage factors influence climate change risk perception, and how can outrage factors be arranged in order of influence on climate change risk perception?
(b) Does political orientation influence the level of climate change risk perception?
(c) Does political orientation interact with outrage factors in guiding climate change risk perception?

## 2. Methods

### 2.1. Data Collection

Embrain, a professional survey agent in South Korea, conducted a nationwide online survey to measure the perceived outrage factors and risk perception regarding climate change. The agent invited 1623 South Koreans based on a quota sampling, and of these, 592 completed the survey (36.5% response rate). Among the respondents, 49.7% (*n* = 294) were male and the average age was 41.9 years old, which is slightly above the average age of the South Korea population (40.2 years). The number of respondents in their 20s, 30s, 40s, 50s, and 60s was 112 (18.9%), 131 (22.1%), 152 (25.7%), 173 (29.2%), and 24 (4.1%), respectively. Our data were roughly representative of the percentage of each age group in South Korea, except for those in the 60s age bracket, who did not have the same level of internet access as the other groups (Table 1).

### 2.2. Measurements

#### 2.2.1. Predictor Variables

Among the 20 outrage factors listed by Covello and Sandman [22], 14 were examined as outrage factors for fine dust with the exclusion of some overlapping and inapplicable factors [36]. Of the 20 outrage factors originally identified by Covello and Sandman, understanding, effects on future generations, victim identity, ethical/moral nature, media attention, and accident history were excluded. This decision was made following the early studies that considered the excluded factors’ overlap with other factors: understanding, effect on future generation, and ethical/moral nature show similarity with familiarity, effects on children, and trust, respectively [36,43]. With regards to media attention and accident history, we consider them as inappropriate because media attention to a risk can be measured more correctly by content analysis of media coverage of climate change. Accident history is also problematic when considering that the global risk is an unprecedented danger to which people have never experienced a similar accident in the past.

In addition, we further excluded the factors benefit, catastrophic potential, and dread. The latter two factors were removed because these factors themselves imply danger. This means that perceiving the two factors indicates perceiving danger, which is similar to risk perception. If a predictor conceptually overlaps with a variable to be predicted, and the former logically signifies the latter, it does not make much sense to examine an association between them. It would be reasonable to expect that if an individual perceives a risk as having a catastrophic potential, he or she may feel that this risk can cause severe damage, which can lead to a higher risk perception. A similar effect is expected when an individual feels that a risk is dreadful.

Measurement of the outrage factor benefit would involve asking whether or not one thinks climate change has a benefit, which is essentially redundant in relation to the question concerning the negative effects of climate change in that the answer to one is likely to be consistently positive and to the other, consistently negative. If one thinks climate change has negative effects, he or she is likely to hold a higher risk perception. As such, measurement of benefit, along with catastrophic potential and dread, can overlap with the measurement of risk perception itself. A total of 11 outrage factors were thus examined in this study.

We used the detailed definition and example of each outrage as described by Covello and Sandman [22] to measure each outrage factor. For example, voluntariness involves involuntary risks (e.g., exposure to chemicals) that are perceived as more dangerous than voluntary risks (e.g., mountain climbing or sunbathing). Controllability also involves uncontrollable risks (e.g., release of toxic chemicals by industrial facilities) that are regarded as more dangerous than controllable risks (e.g., driving an automobile or riding a bicycle). The definitions and examples were also used to develop measurements for outrage factors in food risks [35,43].

Similarly, we used the definitions and examples to develop measurements of outrage factors in climate change. For example, the respondents were asked to rate the degree of agreement with the following statements on a 7-point Likert scale (1 = not at all; 7 = very likely): “A risk from mountain climbing or smoking is one to which individuals are exposed voluntarily. Climate change is likely to be a voluntary risk.” In the case of controllability, the respondents rated the following statement on a 7-point Likert scale (1 = not at all; 7 = very likely): “Risk from climate change is likely to have a similar characteristic to the risks from driving or bike riding that are under our control.” The same pattern of utilizing the original definitions and examples was continued in the other statements using a 7-point Likert scale (1 = not at all; 7 = very likely): “hurting while doing housework” (familiarity), “affects those in a lower socio-economic status” (fairness), “hurts children and future generations” (effect on children), “my family and myself are likely to be affected more than others” (personal stake), and so on. The original values for voluntariness, controllability, and familiarity were reverse-coded such that involuntariness, uncontrollability, and unfamiliarity were represented by a higher score. The full statements used to assess outrage factors are listed in Table 2.

Regarding political orientation, we asked the respondents to rate themselves on a 7-point Likert scale (1 = conservative; 4 = neutral; 7 = liberal). Korean conservatives strongly support national economic growth through a solid partnership with the U.S. [44], and prioritizing economic growth over other values such as democratization had been one of the major characteristics of the military leadership for more than 30 years by Park Chung-hee and Chun Doo-hwan, who took power by military coups. Less environmental and more economic orientation, therefore, would represent South Korean conservatives. South Korean liberals would emphasize reunification with North Korea, equal rights of socio-economic minorities, and equally prioritizing collective assets such as nature as much as economic growth, compared to conservatives. Given the polarization of news consumption reported in the United States [45] and South Korea [46], the perceived political orientation of the news media the respondents used was also measured by asking them to mark the political orientation of the media that they frequently consume (1 = very conservative; 4 = moderate; 7 = very liberal). The internal consistency for the two measurements had a Cronbach’s α value of 0.74.

#### 2.2.2. Control Variable

We controlled for additional factors, including self-efficacy, knowledge, and media use, considering their influences on risk perception observed in the previous studies [7,9,34]. For self-efficacy, which refers to people’s belief in their capacity to execute behaviors necessary to produce specific performance attainments [47], the respondents rated the following two positions on a 7-point Likert scale (1 = least likely; 4 = moderate; 7 = very likely): “I believe that my activities will influence climate change,” and “My coping behaviors will stimulate a similar response from others” (Cronbach’s *α* = 0.74). Knowledge was measured with five true-or-false questions concerning sources of methane gas, the impact of ozone on climate change, South Korea’s responsibility for climate change mitigation, the rise in global temperatures, and the location of the headquarters for a new U.N. climate agency. The number of correct answers represented the knowledge score.

One question and a statement were used to measure media use as follows: “How many days do you use news media per week?” and “Using news media is an important daily event of mine” (1 = Not at all; 4 = moderate; 7 = very true; Cronbach’s *α* = 0.66). We also controlled for age, gender, education, income, and the size of the respondent’s residential area (i.e., metropolitan, small-/medium-sized cities, and rural communities).

#### 2.2.3. Dependent Variable

Risk perception involves evaluating the probability and severity of a risk [48]. Two questions were asked to capture these two dimensions: “As the current situation continues, how likely is climate change to damage our nation?” (probability, 1 = never; 7 = very likely), and “How severe are climate change consequences for Korea?” (severity, 1 = not serious at all; 7 = very serious). We also asked a question regarding the degree of general concern: “What does climate change mean to you?” For the last question, a response of “7” on the Likert scale indicated, “I worry about it very much,” while “1” indicated “I do not worry about it at all.” There was internal consistency among the four items (Cronbach’s *α* = 0.87).

### 2.3. Analysis

Multivariate regression analyses were conducted with a comprehensive model to predict climate change risk perception. A total of 11 outrage factors were used as major factors in addition to other factors examined in earlier studies (self-efficacy, knowledge, political orientation, and media use), while controlling for several demographic factors (age, gender, income, education, and type of residential area). Taking a stepwise approach, we placed multiple interactions into the model to investigate all possible interactions between outrage factors and other factors.

To reduce multi-collinearity in the interactive regression model, all factors for which an interaction effect was tested were mean-centered [49]. The Interplot tool in the R software package [50] was used to visualize the direction of the interaction. Changes in the coefficients of the outrage factors induced by significant moderators were also plotted.

## 3. Results

### 3.1. Descriptive Statistics

Participants believed that the probability of climate change damaging the nation was higher than the level of “somewhat high” represented by the score “5 ”(M = 5.33, SD = 0.89; *t* = 8.96; *p* < 0.01) and the perceived seriousness of the damage was greater than “somewhat serious” (“5”; M = 5.21, SD = 0.93 *t* = 5.59; *p* < 0.01). When asked how much they worry about climate change, their concerns were greater than “somewhat worry”, which is represented by “5” (M = 5.31, SD = 0.94).

**Figure 1:**
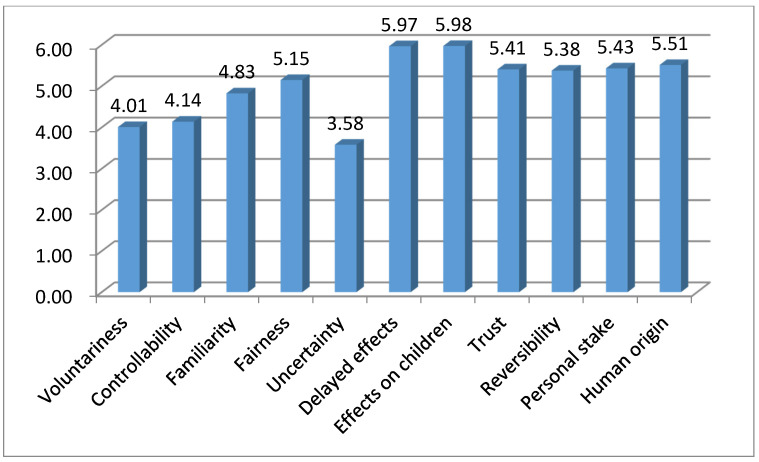
The perceived outrage factors regarding climate change.

The greatest perceived outrage factor was “effects on children” (M = 5.98, SD = 0.98), followed by “delayed effects” (M = 5.97, SD = 1.09), and “human origin” (M = 5.51, SD = 1.12). Being unfamiliar (M = 3.17, SD = 1.50), uncontrollable (M = 3.86, SD = 1.58), involuntary (M = 3.98, SD = 1.56), and uncertain (M = 3.58, SD = 1.443) were considered less serious (Figure 1). The differences were significant in an analysis of variance (ANOVA) test (F = 796.0; *p* < 0.01).

### 3.2. Perceived Outrage Factors and Risk Perception

The first question addressed which outrage factors were significant and the order in which they influence climate change risk perception. Before testing the main effect of outrage factors, we first examined how the control factors were associated with risk perception levels. Among the various factors added to the model to predict risk perception, self-efficacy (*b* = 0.11; *t* [561] = 3.47; *p* < 0.01) was a significant factor in that those who had greater self-efficacy had a higher risk perception of climate change (Table 3).

The main effects of perceived outrage factors on climate change risk perception were found for the risk characteristic of personal stake (*b* = 0.15; *t* [561] = 3.99; *p* < 0.01) and effects on children (*b* = 0.16; *t* [561] = 3.38; *p* < 0.01). Familiarity (*b* = 0.08; *t* [561] = 3.42; *p* < 0.01) and fairness (*b* = 0.04; *t* [561] = 2.16; *p* < 0.05) were also influential. The effect of trust almost reached statistical significance (*b* = 0.07; *t* [561] = 1.81; *p* = 0.07). Participants who perceived that climate change would have a greater influence on themselves and their family than it would for others showed higher risk perception. Those who perceived a greater influence of climate change on children showed higher risk perception than those perceiving a lesser influence on children. Climate change risk perception was also heightened when the respondents thought of climate change as an unfamiliar risk, or when they considered climate change as an aftermath of unfair processes, compared to those considering climate change to be familiar or who recognized little unfairness involved in climate change. Although the perceived characteristics of “delayed effects” and “human origin” were the second- and third-most intense outrage factors, they did not significantly affect climate change risk perception.

### 3.3. Political Orientation and Outrage Effects

Our next research question concerned whether the political orientation of the respondents influenced the level of climate change risk perception. Unlike an earlier finding for a study conducted in the United States, the political orientation of South Koreans was not significantly associated with climate change risk perception when tested in a multiple regression model. When the respondents were divided into three groups (conservatives, moderates, and liberals) based on their orientation score, the analysis of variance test also showed no significant difference in risk perception.

**Figure 2:**
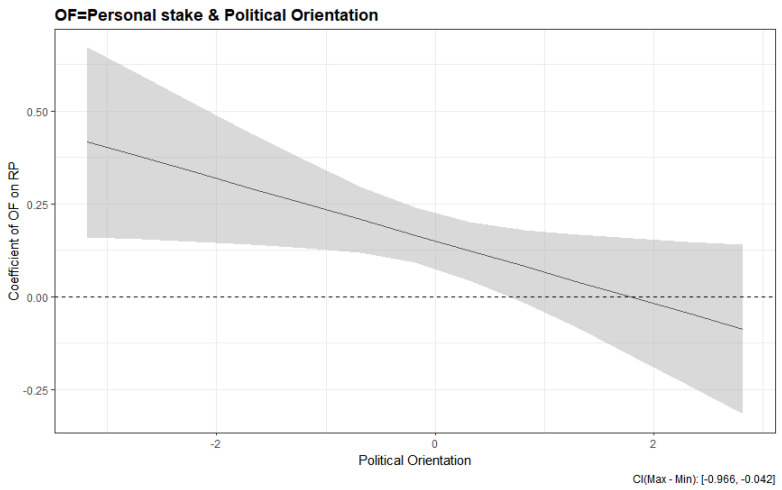
The coefficients of perceived outrage factors (OF) on climate change risk perception (RP) moderated by political orientation.

The final question in this study concerns whether the magnitude of outrage effects vary according to political orientation, signifying an interaction of outrage factor and political orientation in guiding climate change risk perception. We found that political orientation did indeed interact with the risk characteristic of personal stake, such that the effect of perceived personal stake on risk perception was greater for conservatives relative to liberals (*b* = −0.08; *t* [561] = −2.15; *p* < 0.05, Figure 2). The outrage effect of unfairness almost reached a significant level of influence in that liberals who thought that climate change is likely to affect those of a lower socio-economic status tended to show a higher risk perception than conservatives who had a similar opinion (*b* = 0.04; *t* [561] = 1.90; *p* = 0.06).

## 4. Discussion and Conclusions

In this study, a comprehensive set of individual outrage factors and political orientations was examined in terms of their effect on climate change risk perception. Four outrage factors, personal stake, effects on children, familiarity, and fairness, were shown to be directly associated with climate change risk perception. In particular, if climate change was perceived as being influential to individuals and their families, a higher risk perception was observed. When the respondents felt unfamiliar with climate change risk, or when they thought climate change influences children or unfairly affects those of a lower socio-economic status relative to other groups, the risk perception was also heightened. In addition, this outrage effect was more salient to conservatives than liberals in terms of the outrage factor of personal stake.

These findings have several notable implications in the context of risk perception studies regarding climate change as well as on the environment in general. First, we identified characteristics of climate change that heighten risk perception to define significant outrage factors that are specific for climate change. The climate change risk perception of an individual can be influenced not only by a generically positive or negative affect toward the global climate risk, but also by the specific features of climate change they perceive. The results of this study specified these influential features and expand on earlier knowledge regarding the influence on climate change risk perception in the affective dimension [11].

Second, the specified features of climate change are consistent with the characteristics that are influential for the risk perception about the environment in general, suggesting a general tendency in risk perception dynamics regarding both climate change and generic environmental issues. The significant effects of “personal stake” and “effects on children” can belong to *impacts on humans* [23] or *impacts* [25], both of which were extracted from factor analyses of the influences on general environmental risk perception. Familiarity can be regarded as understandability [25], particularly when considering that we originally removed the outrage factor of “understanding” based on its similarity to familiarity. Therefore, the current findings are consistent with those of previous studies of environmental risk perception, suggesting that risk perception is likely to increase when people recognize its personal consequences and when they feel unfamiliar with the risk, whether climate change risk or environmental risk in general is being considered.

Third, it should also be noted that the current findings provide not only a better understanding of climate change risk perception, but also have practical implications. The salient outrage effects of personal stake and effect on children indicate that emphasis on, or framing, the negative impacts of a risk to individuals and children would be an efficient strategy for communicating information about the risk of climate change to the public. In this respect, results of this study demonstrate that studying individual characteristics of a risk could provide a basis for developing coping strategies to respond to environmental threats [27].

Another important finding of this study is that outrage factors interacted with political orientation, which moderated the effect of the perceived characteristics of personal stake. When Korean conservatives recognized climate change as being more influential to their family and themselves, they showed a higher risk perception than when Korean liberals felt this personal stake. In addition, the interaction between the outrage factor of fairness and political orientation almost reached a statistically significant level such that liberals in South Korea were more likely to be affected by the outrage factor of fairness in perceiving climate change risk than the conservatives. In the United States, the mindset of liberals is regarded as being similar to that of a “nurturing mother,” whereas that of conservatives is similar to a “strict father” who wants his children stand up for themselves [51]. Based on this mindset, liberals in the United States have a greater concern about environmental issues than conservatives do [40,41,42]. Even though value-based political orientation did not directly influence climate change risk perception by South Koreans, conservatives in South Korea tended to care more about how they and their family would be affected by climate change. This finding adds empirical knowledge to previous studies that investigated the role of political orientation in guiding climate change risk perception [40,41,42] and demonstrates an additional type of influence that political orientation can have on climate change risk perception.

This study has some limitations. The significant interaction of political orientation with personal stake, but not with other outrage factors, was not explained in the context of any established theoretical perspective. Taking an individual approach to each outrage factor, or risk characteristic leaves various possibilities open for investigation based on different types of risks, multiple outrage factors, and various moderators. Thus, identifying a consistent pattern of interaction across different types of risk may represent a new line of research on risk perception. Based on the results from interaction studies, we can develop an interaction typology that can be connected to a theoretical perspective that will help increase our knowledge of risk perception and risk communication not only in the context of climate change, but on other topics in general.

In addition, it should be noted that even though various risk characteristics were conceptually grouped under the labeling of outrage factor and their influence on risk perception were confirmed, outrage itself as an emotive response was not substantiated with any type of measure, whether physiological (e.g., heart rate, skin conductance) or self-reported [52]. Risk perception studies in the psychometric paradigm [53] can actually evolve into the next level by assimilating this measurement study of outrage.

## Figures and Tables

**Table 1:** The demographic information of the survey participants.

Demographic Factors		Percent (%)
Gender	Male	49.7
	Female	50.3
Age (M = 41.9)	19–29	18.9
	30–39	22.1
	40–49	25.7
	50–59	29.2
	60≤	4.1
Education	Middle≥	**0.7**
	High	18.7
	College	68.4
	Graduate≤	12.2
Income (won)	≤1 million	2.4
	1 m ≤–< 3 m	23.8
	3 m ≤–< 5 m	39.0
	5 m ≤–< 7 m	23.2
	≥7 m	11.7

**Table 2:** The statements utilized to measure perceived outrage factors and source of outrage from which the statements were developed.

Outrage Factors	Source of Outrage	Statement to Rate the Respondents’ Perceived Outrage Factor
Voluntariness	Involuntary or imposed activities	A risk from mountain climbing or smoking is the one to which individuals are exposed voluntarily. Climate change is a risk with this characteristic of voluntariness.”
Controllability	Activities viewed under others’ control	“Risk from climate change has a similar characteristic to the risks from driving or bike riding that are under our control.”
Familiarity	Unfamiliar activities	“Risk from climate change is not so much unfamiliar, but rather it seems to be as familiar as hurting while doing house work”
Fairness	Unfair activities	“Risk from climate change is likely to affect those in a lower socio-economic status”
Uncertainty	Unknown activities or uncertain risks	“Scientific evidence supporting the danger of climate change is uncertain”
Delayed effects	Long latency periods between exposure and adverse effects	“Climate change will cause greater danger to the entire world even though it does not cause a serious problem at the moment”
Effects on children	Activities that specifically put children at risk	“Climate change will especially hurt children and future generations”
Trust	Individuals, institutions, or organizations lacking in trust and credibility	“Climate change is caused by the incompetent government ignoring its obligation of protecting the environment, and companies’ illegal or immoral economic activities”
Reversibility	Irreversible adverse effects	“Damage by climate change is irreversible”
Personal stake	Activities that place one personally/directly at risk	“Climate change is a risk that my family and myself are likely to be affected more than others”
Human vs. natural origin	Risks by human action, failure or incompetence	“Climate change is a risk of modern society caused by human activities more than it is by natural phenomena”

Note: Source of outrage is based on Covello and Sandman’s (2001) definitions of outrage factors.

**Table 3:** The influences on the South Koreans’ climate change risk perception.

Factors	Β (S.E.)	*t*	Β (S.E.)	*t*
Demographic factors				
Age	0.000 (0.003)	−0.069	−0.001 (0.003)	−0.043
Gender	0.026 (0.055)	0.472	0.051 (0.056)	0.384
Income	0.021 (0.013)	1.645	0.018 (0.013)	1.387
Education	−0.032 (0.049)	−0.662	−0.040 (0.049)	−0.815
Residential area	0.003 (0.005)	0.47	0.003 (0.006)	0.461
Perceivers’ character				
Self-efficacy	**0.115 (0.032)**	3.581 **	**0.112 (0.032)**	3.474 **
Knowledge	−0.025 (0.029)	−0.855	−0.032 (0.029)	−1.106
Media use	0.036 (0.024)	1.504	0.035 (0.024)	1.464
Outrage factors				
Voluntariness	0.021 (0.020)	1.064	0.024 (0.020)	1.208
Controllability	−0.030 (0.021)	−1.44	−0.029 (0.021)	−1.39
Familiarity	**0.081 (0.023)**	3.593 **	**0.079 (0.023)**	3.421 **
Fairness	0.043 (0.019)	2.249	**0.042 (0.019)**	2.164 *
Uncertainty	−0.003 (0.021)	−0.148	−0.002 (0.021)	−0.082
Delayed effect	0.048 (0.036)	1.336	0.047(0.036)	1.288
Effects on children	**0.176 (0.045)**	3.886 **	**0.155 (0.046)**	3.380 **
Trust	0.057 (0.037)	1.535	0.069 (0.038)	1.805
Reversibility	−0.006 (0.025)	−0.245	0.006 (0.026)	0.212
Personal stake	**0.158 (0.037)**	4.288 **	**0.149 (0.037)**	3.993 **
Human origin	0.048 (0.033)	1.471	0.050 (0.033)	1.52
Political Orientation (PO)	−0.031 (0.028)	−1.121	−0.025 (0.029)	−0.853
Interaction with PO				
Personal stake			**−** **0.084 (0.039)**	**−** **2.147** *
Adjusted R^2^	0.397		0.400	

Note: In the case of interaction, only significant cases were bolded. * *p* < 0.05; ** *p* < 0.01.
